# The Influence of Serum Sample Storage Conditions on Selected Laboratory Parameters Related to Oxidative Stress: A Preliminary Study

**DOI:** 10.3390/diagnostics10010051

**Published:** 2020-01-19

**Authors:** Lilla Pawlik-Sobecka, Katarzyna Sołkiewicz, Izabela Kokot, Aleksandra Kiraga, Sylwia Płaczkowska, Agnieszka Matylda Schlichtinger, Ewa Maria Kratz

**Affiliations:** 1 Department of Nervous System Diseases, Faculty of Health Sciences, Wroclaw Medical University, 51-618 Wroclaw, Poland; lilla.pawlik-sobecka@umed.wroc.pl; 2 Department of Laboratory Diagnostics, Faculty of Pharmacy, Wroclaw Medical University, Borowska Street 211a, 50-556 Wroclaw, Poland; izabela.kokot@umed.wroc.pl (I.K.); a.kiraga94@gmail.com (A.K.); ewa.kratz@umed.wroc.pl (E.M.K.); 3 Diagnostics Laboratory for Teaching and Research, Faculty of Pharmacy, Wroclaw Medical University, Borowska Street 211a, 50-556 Wroclaw, Poland; sylwia.placzkowska@umed.wroc.pl; 4 Faculty of Physics and Astronomy, Institute of Theoretical Physics, University of Wroclaw pl. M. Borna 9, 50-204 Wroclaw, Poland; a.m.schlichtinger@gmail.com

**Keywords:** blood serum, storage of biological material, cyclic thawing, oxidative stress parameters

## Abstract

The present work aims at accessing the stability of biological material stored for diagnostic and scientific purposes. The influence of the temperature, storage time, and cyclic thawing on concentration stability of selected oxidative stress parameters in human serum was investigated. The study group consisted of 20 serum samples collected from healthy volunteers aged 18–52. The parameters whose reference ranges were not determined and to which validated determination methods did not correspond were examined by manual methods (FRAP and AOPP). Automatic methods were used to determine routine laboratory tests (albumin, total protein, bilirubin, uric acid) using the Konelab 20i^®^ analyzer. The samples were stored at various temperatures (room temperature, 4 °C, −20 °C, −80 °C) for max 6 months and were subjected to cyclic thawing at 1 month intervals. In order to check whether any differences between the concentrations of the studied parameters existed when the samples were stored in various conditions, the paired Student t-test or Wilcoxon test and comparison to desirable bias were applied. Based on the obtained results, it was found that the temperature and time of serum sample storage significantly affected the stability of the analyzed parameters and determined different shelf lives of serum samples for oxidative stress examination. Therefore, continuing the investigation concerning the impact of storage conditions on various serum parameters seems justified due to the discrepancy between the individual results obtained by different researchers and the inconsistencies between the results of scientific research and the applicable recommendations.

## 1. Introduction

Results of clinical laboratory tests constitute an integral part of clinical medicine and are necessary to make decision about patients’ treatment [1]. The growing number of available laboratory tests, research projects, and biobanking procedures gives the opportunity to explore the previously unknown areas of medicine; on the other hand, it is a source of difficulties in interpretation [2]. It is extremely important to ensure the highest quality, comparability, and reproducibility of the analyses conducted, which result in the obtaining of reliable results.

Numerous studies documented that the preanalytical phase is very important and very sensitive to any mistakes, most of which are made in this phase, primarily because of the difficulty in achieving standardized procedures for sample collection and storing [3,4,5,6,7]. Most clinical laboratories standardize the preanalytical phase by controlling and monitoring the preanalytical variables (e.g., specimen collection and handling), thus reducing the magnitude of the errors or inaccuracies in the obtained laboratory test results associated with these parameters. Nevertheless, the scale of inaccuracy of the preanalytical variables as well as their influence on the results of laboratory tests remain significant [8].

An appropriate method of sample storage is a procedure that will not significantly affect the stability of the determined parameters [9]. The literature provides little information about the impact of sample storage conditions, such as time and temperature, and the stability of the concentration of oxidative stress parameters, in particular those evaluating total oxidative or antioxidant status. Additionally, in the case of better-known parameters, the recommendations regarding proper storage of samples are not completely defined [10]. The study conducted by Jansen et al. [11,12] documented that storage time and temperature might affect the stability of oxidative stress biomarkers, which have to be analyzed after long-term storage of serum samples. The authors of a study from 2015 [11] concluded that storage at −80 °C is advised in order to maintain a reliable assay outcome when serum samples have to be stored for longer periods. In 2017, Jansen et al. [12] analyzed the stability of three biomarkers reflecting oxidative stress: reactive oxygen metabolites (ROM) for hydroperoxides, total thiol levels (TTL) for the redox status, and biological antioxidant potency (BAP) for the antioxidant status, which were investigated at several time points during a period of 60 months of storage at −20 and −80 °C. Among other things, they concluded that serum samples for the analysis of the above set of oxidative stress biomarkers can be stored for up to 60 months at −80 °C. However, ROM and BAP can also be stored at −20 °C during this period [12].

Apart from AOPP (advanced protein oxidation products) and FRAP (ferric reducing antioxidant power), which are considered to be markers of oxidative–antioxidant balance, some other parameters that we studied may also participate in oxidative stress. One of them is blood plasma albumin, which has a wide variety of functions in the body (e.g., it is responsible for maintaining colloido–osmotic pressure and transports a variety of substances) [13]. It is able to react with free radicals, peroxides, and hypochlorite, which protects other components with fully essential functions from damage [14]. The next parameter, bilirubin, is classified as a physiological hydrophobic antioxidant. In the form associated with albumin, it protects linolenic acid, also associated with this protein, against peroxidation, while in the conjugated form with glucuronide, it can react with hypochlorite [15]. Uric acid also plays an important role in the antioxidant defense of the body, because it has the ability to bind iron ions and react with oxidizing agents. Uric acid is considered to be the most important substance responsible for total antioxidant capacity of blood plasma [16], being a powerful scavenger of free radicals, and provides ∼60% of free-radical scavenging capacity in plasma [17].

Taking into account the above information about the oxidative stress parameters analyzed in the context of time and temperature of sample storage, there is a need to learn about the factors of storage conditions, which cause changes in the value of the concentration of determined parameters. Such knowledge could significantly contribute to the improvement and unification of the procedures for biological material banking. The originality of our research consists in the assessment of the usefulness and stability of biological material, taking into account its handling in the pre-analytical phase. Biological samples for scientific research projects can be collected differently and stored in different conditions. Currently, depositing samples of biological material in biobanks, i.e., institutions collecting and storing various biological material according to strictly defined procedures, constitutes a good practice. In order to continuously improve the quality of laboratory tests, standardization of parameter determination procedures is introduced at all stages of the analytical process. Scientific studies documented that the majority of irregularities resulting from mistakes are made in the pre-analytical phase, including storage of test samples. The literature provides little information on the impact of biological material storage conditions, such as time and temperature, and on the stability of oxidative stress parameters, particularly those assessing total oxidative or antioxidative status.

Therefore, the aim of this study was to determine the influence of storage time and temperature on the results of oxidative stress parameters in sera samples from healthy volunteers. For this purpose, we examined the analytical stability of the analyzed constituents following numerous freeze–thaw and short- and long-term storage in various temperatures at multiple time points. Our aim was to check whether there was a correlation between the suitability for determination of selected oxidative stress parameters in biological material such as human serum and sample storage conditions.

## 2. Materials and Methods

Each of the 33 adult volunteers recruited for our project completed a questionnaire about their current status of health (including inflammation, infections, and chronic diseases). Moreover, on the day of blood collection, all volunteers had measured completely blood count parameters (5-Diff analyzer) as well as additional biochemical parameters (inter alia, concentrations of glucose, cholesterol, HDL, LDL, triglycerides, iron, and CRP-hs). Based on the obtained results and information included in the questionnaire, twenty healthy adult volunteers for our project were selected. The study group consisted of 10 males and 10 females (age range 18–52 years). Thirty milliliters of venous blood was collected from each volunteer. All samples were collected after informed consent had been provided by the subjects. The study No ST.D160.18.009 was approved by the Wroclaw Medical University Bioethics Committee (No KB-136/2018). Blood samples were allowed to clot for 30 min at room temperature (RT), and then they were centrifuged at 2000× *g* for 10 min at room temperature in order to obtain serum. On the day of sampling (T_0_), the initial concentrations of selected oxidative stress parameters in the serum samples were determined. The samples tested were divided into three main groups and each of them was stored under specific conditions and for a specific period of time (Table 1). Each patient’s serum sample was divided into 24 separated aliquots (3 portions were stored at RT, 3 at 4 °C, 5 at −20 °C, 5 at −80 °C, 4 at −20 °C/T/F (thawing and freezing), 4 at −80 °C/T/F) in which the determination of the studied parameters was verified only once. The first group comprised samples that were stored simultaneously at room temperature and at 4 °C (fridge temperature, FT). The analyzed parameters were determined in all samples 24, 48, and 120 h after blood collection. The other two batches of collected serum were frozen, one at −20 °C, and the second at −80 °C, in order to carry out the mentioned determinations after 1, 2, 3, 4, and 6 months from the date of collection. A part of the pool of samples frozen at −20 °C and −80 °C was subjected to a process of cyclic thawing and freezing (R1, R2, R3, R4). All patients’ samples subjected to cyclic temperature changes (4 aliquots for −20 °C and 4 aliquots for −80 °C) were thawed, but only one of the samples stored at −20 °C and one from those stored at −80 °C were used to perform the analysis; the remaining samples were frozen again for further testing. This procedure resulted in the opportunity to analyze the possible effect of changes in temperature during sample storage on the analyzed parameters. We evaluated both statistical and clinical comparisons. Median or mean values of the determined parameters obtained in subsequent points of the study procedure were compared with T_0_ concentrations (reference point used for statistical analysis of differences between the studied parameters, depending on the changed conditions of sample storage). Clinical comparison was conducted according to the desirable bias (DB) thesis recommended by Ricos et al., first published in 1999 [18] and updated in 2014 [19]. Bias is the difference between the mean of the test results and the reference value [20]. For the purposes of our determination it was the initial concentration, T_0_. According to this assumption we adopted the desirable bias published by Ricos et al. [19] as the limit for the comparison of the mean concentrate percentage change in every study point for each analyzed parameter. If the value exceeded DB, we concluded that a sample stored in particular conditions became useless for laboratory purposes.

The manual methods used by us for the purposes of FRAP and AOPP determination have not been validated so far; therefore, we assessed their shelf life according to the guidelines [21], considering DB for new methods and taking into account the maximum deviation, which is equals 10%.

The concentrations of albumin, total protein, bilirubin, and uric acid were determined using the Konelab 20i^®^ (Thermo Scientific, Ratastie 2, Vantaa, Finlandia) biochemical analyzer. The test used for the determination of albumin concentration was based on the reaction of albumin with bromocresol green (BCG). As a result of the reaction, a colored product formed, and the absorbance was measured at 600 nm [22]. The biuret method was used for the determination of total protein concentration [23]. Briefly, protein and cupric ions in alkaline solutions formed a colored complex, and the absorbance of the formed complex was subsequently measured at 540 nm. The method employs EDTA as a chelating and stabilizing agent for cupric ions [24]. Bilirubin reacts with dinitrogenized sulphanilic acid, which results in formation of an intense dia blue dye product. The intensity of the color is proportional to the total bilirubin concentration in the test sample, and the absorbance was measured at 575 nm [25]. The next parameter, uric acid, is oxidized to allantoin by uricase during the measurement. The generated hydrogen peroxide reacts with 4-aminoantipyrine (4-AAP) and *N*-ethyl-*N*-(hydroxy-3-sulfopropyl)-m-toluidin (TOOS), forming a blue–violet product. The absorbance of the formed colored product of the reaction was measured at 540 nm [26].

The total antioxidant capacity and the concentration of advanced protein oxidation products (AOPP) were determined spectrophotometrically with the use of a UV/Vis spectrophotometer (UV-6300PC, VWR, Geldenaaksebaan 464, Oud-Heverlee, Belgia). The total antioxidant capacity for reduction of excessive free radicals in serum samples was measured by the ferric reducing ability of plasma (FRAP) method with ferric tripyridyltriazine [27]. Reduction of Fe^3+^ to Fe^2+^ at low pH forms a colored Fe^2+^–tripyridyltriazine complex. For these reactions, 500 µL of FRAP reagent (freshly prepared by mixing 25 mL of 300 mM acetate buffer (pH 3.6), 2.5 mL of 10 mM 2,4,6-tripyridyl-*s*-triazine (TPTZ) in 40 mM HCl and 2.5 mL of 20 mM aqueous solution of FeCl_3_·6H_2_O) was mixed with 100 µL of a 1:9 diluted sample (diluted in distilled water). The reaction mixture was incubated for 5 min at 37 °C and then centrifuged at 2000× *g* for 10 min at room temperature. The supernatants were transferred to the micro-cuvettes, and then the absorbance was immediately recorded at 593 nm. The absorbances of the samples were read against a reagent blank. A calibration curve was constructed for the known amounts of Fe^2+^ in the solution (ranging from 0.05 to 0.25 mM Fe^2+^). The concentration of AOPP was measured via a spectrophotometric assay, according to the method of Witko-Sarsat et al. [28]. This method is based on the reaction of AOPP with potassium iodide solution in an acidic environment. Briefly, to 400 μL of serum sample diluted 1:4 in PBS, 20 μL of KI was added, incubated 2 min at RT, and mixed with 40 μL of 10% (*v*/*v*) acetic acid. The absorbance was measured immediately at 340 nm against a blank sample without serum but containing all other reagents. The results were expressed in chloramine T units. A calibration curve was constructed for chloramine T concentrations ranging from 0 to 100 μM. All determinations were made in duplicate.

### Statistical Analysis

Statistical analysis of the obtained data was performed using the Statistica PL software, version 13.3. To verify whether any differences existed between the concentrations of the studied parameters in the samples stored in various conditions, the paired Student *t*-test or Wilcoxon test was applied. The Student *t*-test was used for normal sample distribution, which was analyzed using the Shapiro–Wilk test. In case of a statement of normality the Student t-test was applied; however, if the hypothesis was rejected, the Wilcoxon test was applied. A two-tailed *p*-value of less than 0.05 was considered to be significant.

## 3. Results

**Figure 1:**
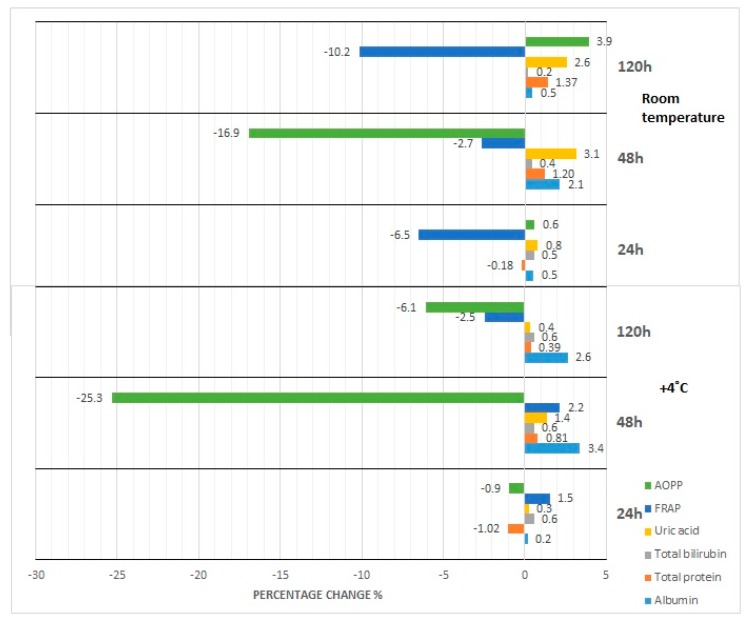
Percentage changes in concentration of selected parameters after storage of serum samples for 24–120 h at room temperature (RT) and 4 °C (fridge temperature, FT), AOPP—advanced oxidation protein products, FRAP—ferric reducing ability of plasma, UA—uric acid, Bil—bilirubin, TP—total protein, Alb—albumin.

**Figure 2:**
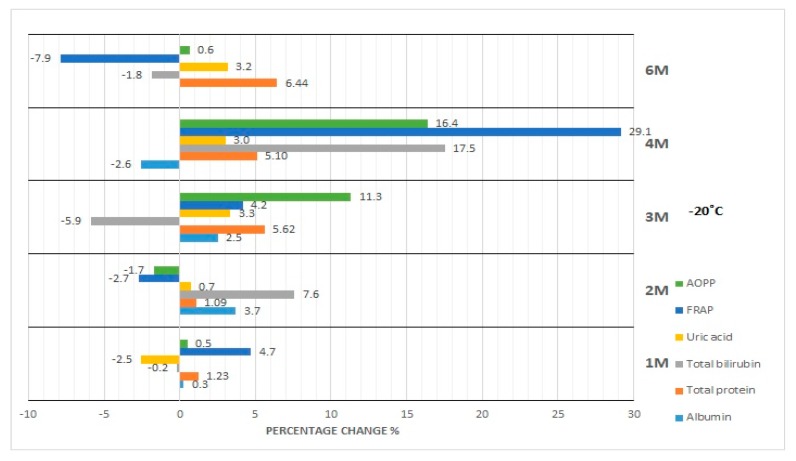
Percentage changes in concentration of selected parameters after storage of serum samples for 1–6 months (1M-6M) at −20 °C. AOPP—advanced oxidation protein products, FRAP—ferric reducing ability of plasma, UA—uric acid, Bil—bilirubin, TP—total protein, Alb—albumin.

**Figure 3:**
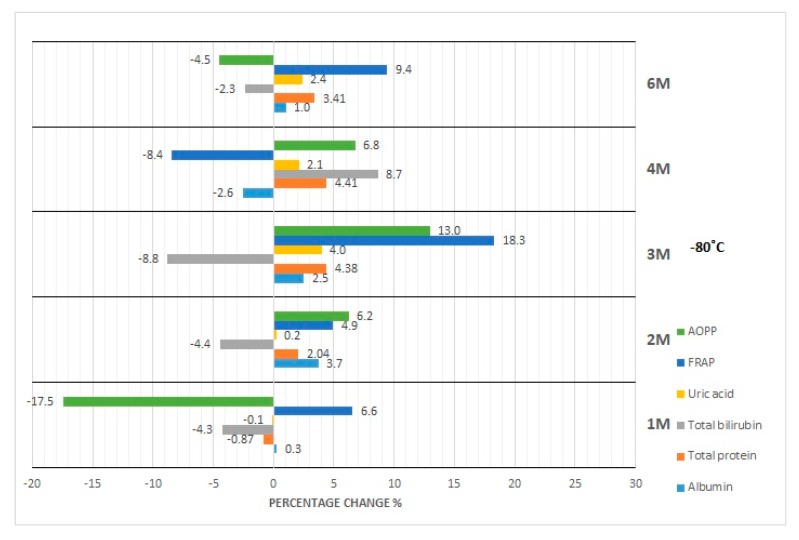
Percentage changes in concentration of selected parameters after storage of serum samples for 1–6 months (1M-6M) at −80 °C. AOPP—advanced oxidation protein products, FRAP—ferric reducing ability of plasma, UA—uric acid, Bil—bilirubin, TP—total protein, Alb—albumin.

**Figure 4:**
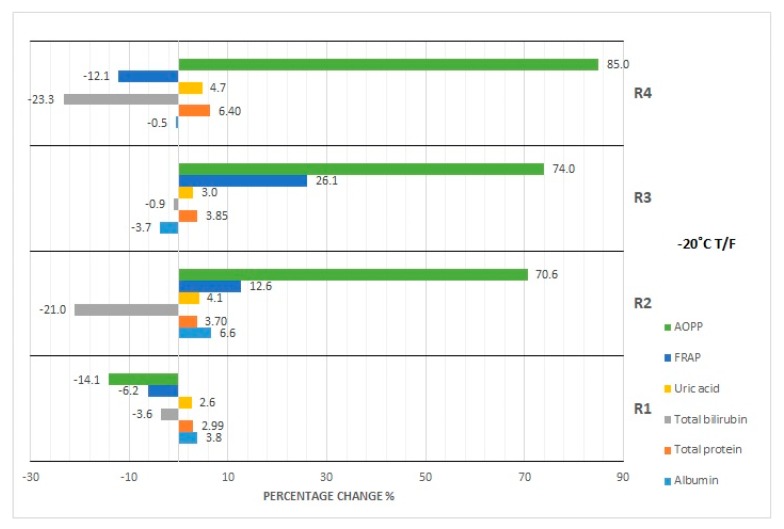
Percentage changes in concentration of selected parameters after cyclic thawing of serum samples (R1-R4) at −20 °C. AOPP—advanced oxidation protein products, FRAP—ferric reducing ability of plasma, UA—uric acid, Bil—bilirubin, TP—total protein, Alb—albumin.

**Figure 5:**
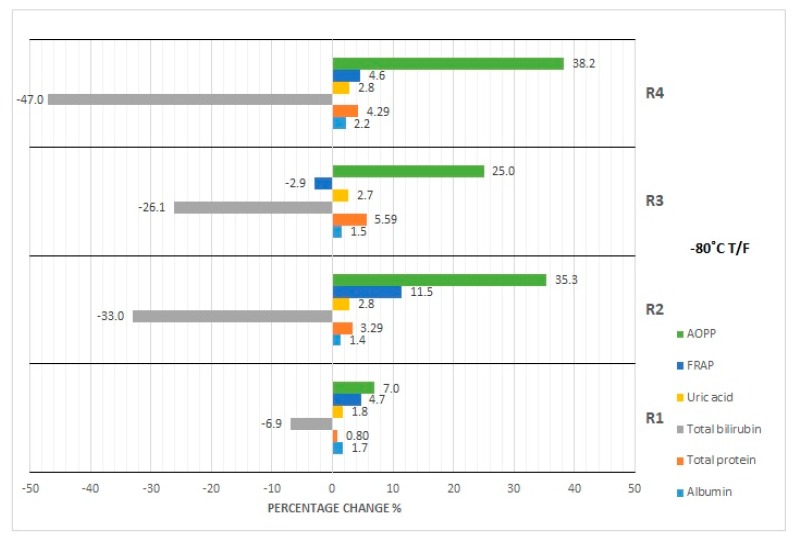
Percentage changes in concentration of selected parameters after cyclic thawing of serum samples (R1-R4) at −80 °C. AOPP—advanced oxidation protein products, FRAP—ferric reducing ability of plasma, UA—uric acid, Bil—bilirubin, TP—total protein, Alb—albumin.

The results of our studies on the stability of laboratory parameters related to oxidative stress following various times and conditions of storage are shown in Table 2 and in Figure 1, Figure 2, Figure 3, Figure 4 and Figure 5.

### 3.1. Albumin Concentration

The concentration of albumin after serum sample storage, both at room temperature and at 4 °C, did not show statistically significant variability up to 48 h (Figure 1); however, the obtained values were burdened with various biases. Clinically significant variability occurred at the 48th hour of sample storage at 4 °C. Moreover, although the desirable bias (1.43%) was exceeded at the 48th hour of sample storage at 4 °C as well as at the 120th hour of storage at RT and 4 °C, the corresponding *p*-value was exceeded when a serum sample had been stored for 120 h at RT and 4 °C. In the case of the storage of serum samples at −20 °C, clinically significant variability was observed from the first month; however, statistically significant variability occurred in the first, third, and sixth month (Figure 2). Due to the changes in this parameter concentration, when stored for up to 6 months at −80 °C, the sample could only be used during the first month (Figure 3), and after this time the obtained values became useless (both statistically and clinically significant variability). Cyclic thawing of samples stored at −20 °C and −80 °C in both cases caused the occurrence of clinically significant changes since the first thawing (Figure 4 and Figure 5). In turn, when a sample was stored at −80 °C it could be thawed once, as evidenced by the *p*-value.

### 3.2. Total Protein Concentration

In the case of sample storage at room temperature, considering the acceptable biases (1.36%), serum samples could not be stored for longer than 48 h (Figure 1), while the *p*-value was exceeded starting from the 120th hour. During sample storage at 4 °C, neither clinically nor statistically significant variability occurred (Figure 1). Human serum stored at −20 °C was not useful for the examination of total protein concentration in the third month of storage (Figure 2), because clinically and statistically significant variabilities occurred. In turn, our results showed that samples stored at −80 °C could only be used in the first month of their storage (Figure 3), taking into account clinically and statistically significant variabilities. The material, which was stored at −20 °C, could not be used even after one thawing (Figure 4), because of the occurrence of statistically and clinically significant variabilities observed at each study point. The serum samples, which were stored at −80 °C, could not be used for the determination of total protein concentration even after only one thawing (Figure 5), because the *p*-value was exceeded after the first thawing, while clinically significant variability occurred after the second thawing.

### 3.3. Bilirubin Concentration

Serum samples for bilirubin examination could not be stored at room temperature even for only 24 h because of statistically significant variability, whereas desirable bias (8.95%) was exceeded from the 48th hour of storage (Figure 1). However, storage of samples at 4 °C made bilirubin examination possible within the period of up to 120 h (Figure 1). Considering the *p*-value, the sample for bilirubin determination could be stored at −20 °C for at least up to 2 months. The desirable bias in this case equaled 8.95%, which was exceeded in the fourth month, and in the sixth month its value decreased again (Figure 2). In the case of *p*-value analysis, sample storage at −80 °C was possible only up to 4 months. On the other hand, changes in bilirubin concentration were the highest in the fourth month (Figure 3), which remains a matter of debate. In the study on the influence of temperature during long-term storage on the storage of serum samples, significant changes were observed only in the fourth and sixth month, while clinically significant changes did not occur at all. Both the statistical test and the analysis of desirable bias indicated that serum samples could not be used for bilirubin examination after the second thawing when stored at −20 °C (Figure 4). Thawing of such material at −80 °C made its use impossible, which was illustrated by the *p*-value at each study point. Looking at the desirable bias, however, it was not exceeded only at the first thawing (Figure 5).

### 3.4. Uric Acid Concentration

The samples for the examination of uric acid concentration could be stored at room temperature for up to 48 h (Figure 1), because the *p*-value was exceeded only after 120 h, and they could be stored up to 120 h at 4 °C (Figure 1). The samples for uric acid determination stored at −20 °C did not show clinically significant changes at each measurement point; however, statistical variability occurred in the first, third, and sixth month of storage (Figure 2). In the case of the serum samples stored at −80 °C, statistical variability occurred in the third and sixth month of storage, but when it came to clinically significant variability, it was the same as it was observed in the case of the samples stored at −20 °C. The analysis of the *p*-value indicated significant differences after the second and third thawing when the sample was stored at −20 °C. On the other hand, the desired bias (4.87%) was not exceeded even once, but the nearest value (4.75%) occurred during the fourth thawing (Figure 4). After thawing of the sample while it was stored at −80 °C, the desirable bias was not exceeded, and it was lower than in the case of storage at −20 °C (Figure 4 and Figure 5). On the other hand, the *p*-value exceeded the critical value each time.

### 3.5. FRAP Concentration

FRAP serum samples could be stored at room temperature for up to 48 h as the desired deviation (10%) was exceeded at the 120th hour of storage (Figure 1). Storing samples at 4 °C made FRAP testing possible within up to 120 h (Table 2). Considering the percentage change in the concentration (10%), the FRAP assay sample could be stored at −20 °C for at least 3 months, whereas storage of the sample at −80 °C was possible for up to 2 months. Cyclic thawing of samples stored at −20 °C and −80 °C caused clinically significant changes in the second thawing cycle in both cases (Figure 4 and Figure 5). Statistically, serum samples could not be used for analysis in any case because the critical *p*-value was always exceeded (Table 2).

### 3.6. AOPP Concentration

In the case of the AOPP concentration determination, serum samples could be stored at room temperature as well as at 4 °C for up to 24 h at most (Figure 1), because at the 48th hour the critical *p*-value was exceeded. The measuring of AOPP concentration in serum samples stored at −20 °C indicated significant variability in the third and fourth month of storage, opposite to the first, second, and sixth months (Figure 2). In the case of sample storage at −80 °C, the *p*-value was exceeded in the first three months. In the fourth and sixth months, significant variability did not occur. Cyclic thawing of the material studied, both after storage at −20 °C and −80 °C, made the application of the sample impossible, because the *p*-value was exceeded (Figure 4 and Figure 5). On the other hand, the observed percentage change of concentration was the highest in the final storage period.

## 4. Discussion

The results of our studies confirmed that the time and temperature of serum sample storage have significant impact on the stability of the tested parameters’ concentration. Knowledge about the influence of human serum storage conditions on the changes observed in the concentration of individual analytes enables correct planning of laboratory tests and measurements of chosen parameters at an optimal time from biological sample collection. When drawing conclusions, it ought to be noted that the shortest period of sample storage indicated in our research will be treated as the shelf life of a specific substance. This is due to the fact that the material collected in this study from individual patients behaves in a different way, which was verified by us, and the results of this examination are presented as: plots of the coefficient of variation for determinations carried out in samples stored in various conditions (Figures S1–S6); correlation coefficient of variation (CV) calculated for specific storage condition with initial concentration (T_0_) (Table S1); changes in concentration of analysed parameters after storage in dependency on time and temperature (Figures S7–S12); available in supplementary materials. 

In our opinion, when it comes to an issue as important as using it to make clinical determinations, one should be sure that the material is reliable. Therefore, in case of any doubts regarding storage time of materials, the result in favor of longer storage will be discarded. In view of the above assumption, in the case of our studies, during storage at room temperature the highest stability was demonstrated by serum sample for clinical determination of uric acid concentration, because desirable bias documented by Ricos et al. [19] was not exceeded until at least 120 h of storage. 

### 4.1. Effect of Storage at Room Temperature and Refrigeration

The lowest stability characterized serum samples when FRAP concentrations were measured. Due to the occurrence of significant variability in the assumed empirical conditions, laboratory examinations ought not to be made 24 h after sample collection. At the same time, for the other parameters (albumin, total protein, bilirubin, AOPP), there is no need for special handling of the collected material within the first 24 h, taking into account its clinical utility determined by DB. Confirmation of the stability of the above parameters present in serum samples in various conditions of biological material storage has been documented by other authors, who analyzed the stability of 81 clinically determined and important parameters [8,30]. The results of the previously conducted studies regarding the optimal conditions of biological material storage for total protein concentration are contradictory. According to Dirar et al. [31] total protein concentration in the serum was stable for 72 h in 4 °C, while Cuhadar et al. [32] observed the first significant changes after 6 h of sample storage, regardless of temperature. Based on our research, it was found that the desirable bias was exceeded after 48 h of serum sample storage at room temperature, while the samples stored at a temperature of 4 °C remained stable for 120 h. This indicates that the temperature of a refrigerator is better for storing the serum for total protein determination. However, it should be noted that statistical analysis was performed apart from the currently accepted clinical guidelines based on Westgard QC (Westgard Quality Control) [18]. The results obtained by us indicate that for uric acid concentration, a significant change (*p* < 0.05) was observed after 120 h of sample storage at room temperature. The above results are in accordance with the results published by Guder et al. [33], according to which the manufacturer’s leaflet for methods used by us was prepared. In the case of other parameters such as total protein and albumin concentration, our results are partially in accordance with the data presented in the aforementioned leaflet. These discrepancies can be explained by the homogeneity of the group of patients examined by us. When it comes to storage at 4 °C, the highest—as mentioned above—stability is demonstrated by uric acid concentration, but also by the concentration of total protein and bilirubin. This is reflected in the Regulation of the Polish Minister of Health—Annex 3 [34], according to which uric acid concentration is a temperature-sensitive parameter, and for preserving of its stability it is recommended to store the serum at 4 °C.

With regard to bilirubin, the results obtained by us are consistent with the results of Kift et al. [35], showing that the concentration of bilirubin in the serum stored at the temperature of the refrigerator remains approximately constant, which confirms the occurrence of photo degradation of this parameter in the case when the tested material is exposed to light, as a result of geometric isomerization and oxidation caused by photons. In addition, the above reactions proceed faster at higher temperatures. Therefore, the serum for determination of bilirubin concentration stored at room temperature decompose faster than that stored in the refrigerator. It is obvious that exposure to light could significantly decrease bilirubin concentration, which was confirmed by the results of the present study. Thus, the samples for determination of bilirubin concentration should be rather stored much shorter on an illuminated countertop at room temperature, than in the dark at 4 °C. The obtained results are consistent with the results of Kift et al. [35], which show that the concentration of bilirubin in the serum stored at refrigerator temperature remains approximately constant. The same authors also concluded that the concentration of total protein increases along with the increase of temperature [35]. The results of our study are consistent with the results of the above-mentioned articles and with the recommendations included in the Annex to the Regulation of the Polish Minister of Health [34], according to which total protein concentration is a parameter sensitive to temperature.

Along with this criterion, DB (10%), FRAP, and AOPP concentrations maintain stability for up to 48 h at room temperature. However, considering the occurrence of significant changes observed by us, FRAP should be marked immediately after material collection, and the serum sample for AOPP concentration determinations should be stored for no longer than 24 h.

### 4.2. Effect of Storage at −20 °C and −80 °C

For the frozen samples stored at −20 °C and −80 °C, considering the desirable bias, the highest stability was again observed for uric acid and bilirubin concentration, although for the second parameter, instability was observed after the 4th month of sample storage at −20 °C. However, considering the *p*-value, the samples should not be stored for more than 2 months. These results are in contradiction with the test manufacturer’s leaflet, on the basis of which the serum bilirubin concentration examination was done, in which there is information that the serum bilirubin concentration is stable when the sample is stored at −20 °C for up to 6 months, much longer than our data indicates. In the study conducted by Cuhadar et al. [36], bilirubin concentration was found to remain stable for up to 3 months of serum storage at −20 °C, in contrast to the Amin and Ahlfors study [37], in which the authors observed a decrease in bilirubin concentration both at −20 °C and at −80 °C after serum sample storage for 2 weeks. Jansen et al. [38] also investigated the method for uric acid and bilirubin determination, testing temperature stability of these parameters, upon storage of serum for 12 months. They selected two or three temperatures most commonly used for storage, that is, −20, −70 (or −80), and −196 °C. The authors observed that both parameters showed no statistically significant differences between the temperatures. They concluded that storage at −20 °C was sufficient to maintain proper assay outcomes for most of the total antioxidant assays, although storage at −70/80 °C was preferred for longer storage times. The confirmation of the stability of the biological material tested by us for the analysis of uric acid concentration, keeping in mind DB (4.87%), may be also found in the studies by Cuhadar et al. [36], Kachhawa et al. [39], or Brinc et al. [40]. It is also in the accordance with the manufacturer’s recommendations that both bilirubin and uric acid concentration remain stable for 6 months after storage of serum samples at −20 °C [25,26]. In our research, the inconsistent occurrence of significant changes in uric acid concentration examined for samples stored at −20 °C is surprising. In the parameters determined by validated methods, albumin concentration was significantly less stable, which contradicts the research of Kachhawa et al. [39] and Cuhadar et al. [36]. In both articles cited above, the authors documented the lack of significant changes in the concentration of this parameter, analyzed during the period of one month of specimen storage at −20 °C, but these results are not in accordance with the standards set out in the Annex to the Regulation of the Polish Minister of Health [34], according to which albumin concentration remains stable for up to 4 months when serum is stored at −20 °C. Significant changes observed by us may be the consequence of an increase in variance caused by exceeding the reference value for albumin concentration in adults (5.0 g/dL) by one of the examined patients. Moreover, the concentration of this parameter exceeds the mean value by more than 2 standard deviations. After the sixth months of storage, the sample is not useful for albumin concentration determination. Therefore, it is safe to assume that the usefulness of serum sample for albumin concentration examination finished during the first month of storage at −20 °C. Our results are in accordance with the results obtained by other authors [36,39].

When assessing the effect of deep freezing temperature on the stability of albumin concentration, we can conclude that significant statistical and clinical changes occurred after 1 month of sample storage at −80 °C. The results incompatible with these observations were presented by Brinc et al. [40], who demonstrated the stability of albumin concentration at −80 °C for a period of 13 months. The discrepancy between our results and the results obtained by Brinc et al. [40] may be explained by the fact that in our study we measured the concentration of albumin in serum samples obtained from adult healthy volunteers, and the authors in their investigations tested blood samples collected from children aged 0–18 years, and these samples were assigned to six categories depending on the age of the patient. Then the samples were pooled and analyzed in this form, whereas in our study the markings were carried out individually for each patient. The utility of our serum samples for markings of total protein concentration after serum storage at −20 °C was observed for up to 2 months. It should be noted, however, that there is significant variability, which excludes the use of serum samples for such investigations in a period of storage at −20 °C longer than two months. In the case of serum storage at −80 °C, utility is shorter (Table 2). The obtained results were not confirmed in the previous reports [36,39,40], according to which total protein concentration remains stable for at least one month after sample storage at −20 °C and up to 13 months at −80 °C. Our results are also not compliant with the recommendations included in the Annex to the Regulation of the Polish Minister of Health [34], according to which the serum samples for the markings of total protein concentration can be stored for up to one year at −20 °C. The results of our study showed that the serum for the measurement of total protein concentration may be stored longer at −20 °C than at −80 °C, which we may attempt to explain by thermodynamic laws. At a reduced temperature, hydrophobic interaction force decreases (the thermodynamic potential of hydration decreases) and the repulsive force inside the molecule increases. As a result, the structure of the protein becomes less compact [41].

Few publications regarding the concentration of oxidative stress parameters determined by manual methods are available. However, Matteucci et al. [42] and Qing et al. [43] analyzed changes in AOPP concentration in serum under various conditions of sample storage time and temperature; the parameters of their analysis (temperature and storage time of sera) were different than the ones tested by us. As far as we know, the effect of serum storage time and temperature on FRAP levels have not been studied before. In our results, AOPP concentration showed greater stability than FRAP. The results of our research are different when compared with the data obtained by Jansen et al. [10,11,12,38]; however, the differences may result from the fact that the authors studied the time and temperature stability of other oxidative stress parameters, not AOPP and FRAP, as we did in our study. It turns out that AOPP concentration is sensitive to temperature changes during serum freezing, which is in contradiction with the information provided by Witko-Sarsat et al. [28], according to which serum stored for more than 6 months at −70 °C can be used for the determination of AOPP concentration. According to the studies by Firuzi et al. [44], storage conditions of biological samples may affect the stability of some oxidative stress markers. The probable cause of these changes may be the generation of oxidation reaction products or the degradation of antioxidants in the stored material. It should be noted that manual tests are at high risk of making a mistake at every stage of the diagnostic process, from reagent preparation, through to the implementation of the test procedure, to the calculation of the final result.

### 4.3. Effect of Repeated Freezing and Thawing

The possibility of using serum samples after repeated thawing is another issue. Considering desirable bias, serum sample for examination of albumin concentration should not be thawed even once if stored at both temperatures (−20 and −80 °C). At the same time, the results of statistical analysis indicate that the serum sample can be thawed once if stored at −80 °C. However, these results should be rejected due to the exceeding of desirable bias. According to the clinical criterion (DB), uric acid concentration becomes the most stable parameter again. Thawing of serum samples for examination of uric acid concentration is possible at all measured points at both storage temperatures (−20 and at −80 °C). According to the statistical analysis, we observed a significant difference in this parameter concentration after the second cycle of thawing if stored at −20 °C, and in every study point after sample storage at a temperature of −80 °C. Perhaps this is related to changes in the pH value; however, to test this hypothesis, further experiments have to be conducted. 

The cyclic thawing of serum samples is not recommended for the determination of total protein concentrations. We observed that significant differences exist between them when the determinations were done in the samples after storage at both −20 and −80 °C. Similar results were obtained for the serum bilirubin concentration if the sample was stored at –80 °C, and they are in line with the results presented in the work by Cuhadar at al. [36], based on which the serum for the determination of bilirubin concentration should not go through more than one thawing cycle after being stored at −20 °C.

As for the other studied parameters, the possible discrepancies between the clinical and statistical analysis may be the result of small differences in the concentration values, which may not be revealed in the case of insufficiently accurate tests. It is also worth mentioning that statistical analysis indicates that samples used for determination of oxidative stress parameters such as FRAP and AOPP should not undergo cyclic thawing processes, both for samples stored at −20 °C as well as at −80 °C. Analyzing only the value of desirable bias, uric acid concentration appears to be most stable in the serum sample during cycles of thawing. Desirable bias is not exceeded in this case even during the fourth thawing. Most likely, this is the effect of a set of biological and chemical changes. Oxidative stress parameters are extremely sensitive to such changes due to their molecular structure [45]. Taking into account all of the above results obtained by us in the study, the fact that despite the observed changes in concentrations of analyzed serum parameters after various times of serum sample storage, subsequent thawing cycles and various storage temperature, as shown in the statistical analysis, the values of the analyzed parameters not exceeded the physiological reference values for the healthy group of patients is interesting and worth mentioning.

### 4.4. Limitations

The weakness of our study includes the necessity to determine the parameters manually (AOPP and FRAP) and the lack of availability of ready-made (commercially available) reagents for their testing, as well as many aspects concerning manual activities during the measurement, which makes it difficult to standardize the above methods, despite the fact that the determinations are carried out strictly in accordance with the commonly applied procedure. In addition, a larger number of subjects in the studied group would allow us to draw more constructive conclusions; therefore, the results of our research can only be considered as indicating the need for further, more advanced analyses.

### 4.5. Strengths

In various variants of serum storage time and temperature, we tested the concentrations of routinely determined parameters (albumin, total protein, bilirubin, uric acid) and parameters mainly examined in scientific projects, thanks to which we obtained information on the usefulness of the biological material we analyzed for the determination of parameters selected by us parameters after sample storage in various conditions. The results obtained by us for the parameters routinely determined in diagnostic laboratories and those determined by manual methods, used mainly in scientific projects, seem significant not only to laboratory diagnosticians stressing the proper handling of the biological material (storage time and temperature), but also to scientists who often in their studies analyze the values of parameters, the concentrations of which can be determined only by manual methods, which are often not validated. The discrepancies between the results obtained by us and the results observed by some other authors should result in researchers’ attempts to study the biochemical mechanisms that occur during the changes of biological material storage.

## 5. Summary

In summary, the results of our study showed that the temperature and duration time of serum storage have significant influence on the stability of concentration of the analyzed oxidative stress parameters. Among them, the highest stability is characteristic of uric acid concentration, while the lowest is characteristic of the concentration of oxidation stress parameters, which were examined by manual methods. Cyclic changes of temperature during samples storage have significant impacts on the stability of all determined parameter concentrations. However, in order to look more closely at this phenomenon, it would be necessary to carry out experiments, both biochemical and related to the physical, especially thermodynamic, effects. The discrepancies between the results obtained using statistical methods and clinical assumptions suggest that the period of samples storage should be as short as possible. It should be taken into account that statistical tests may not fully reflect clinical needs. However, clinical practice requires minimization of the risk that the differences will affect patient’s diagnosis and applied treatment. Thus, despite the fact that for most patients, serum samples can be determined even after a relatively long storage time, one should not forget that there are very sensitive parameters, which require special procedure of storage. The most commonly used solution is the use of information provided by the test manufacturer or generally recognized recommendations. However, our research has shown that they are not always consistent with the observations made in a standard laboratory. This results in the need for careful interpretation of the manufacturer’s information, but at the same time we are aware that it is impossible to carry out such a study as ours for all parameters performed in routine clinical laboratories.

Our experiment confirms that researchers need to use appropriate procedures for biological sample handling from the time of their collection, as it is recommended by good laboratory practice principles, because it has significant impact on obtaining the results of measurements.

## Figures and Tables

**Table 1:** Algorithm of study serum samples storage.

**Storage Period**	**Group I**	**Procedure**			
**24 (h)**	**RT; 4 °C**	Examination of serum			
**48 (h)**					
**120 (h)**					
**Storage Period**	**Group II**	**Procedure**	**Group III**	**Cycle**	**Procedure**
**1 (M)**	**−20 °C; −80 °C**	Thawing and examination of serum	**−20°; −80 °C**	**T/F**	Thawing, and re-freezing of serum
**2 (M)**				**R1**	Thawing, examination and re-freezing of serum
**3 (M)**				**R2**	
**4 (M)**				**R3**	
**6 (M)**				**R4**	

RT—room temperature; T/F—thawing and re-freezing of the same samples; R1,2,3,4—cycles of thawing and re-freezing of the same samples; (h)—hour; (M)—month. Each patient’s serum sample was divided into 24 separated aliquots (3 portions were stored at RT, 3 at 4 °C, 5 at −20 °C, 5 at −80 °C, 4 at −20 °C/T/F, 4 at −80 °C/T/F) in which the determination of the studied parameters was examined only once. All of the patients’ samples subjected to cyclic temperature changes (aliquots for −20 °C and aliquots for −80 °C) were thawed; one sample stored at −20 °C and one stored at −80 °C were used to perform the analysis, the remaining samples were frozen again for further testing.

**Table 2:** Behavior of chosen parameters after serum sample storage in various conditions.

	Time Temperature	T0	24 h RT	48 h RT	120 h RT	24 h FT	48 h FT	120 h FT	1 M −20 °C	2 M −20 °C	3 M −20 °C	4 M −20 °C	6 M −20 °C	1 M −80 °C	2 M −80 °C	3 M −80 °C	4 M –80 °C	6 M −80 °C	R1 −20 °C	R2 −20 °C	R3 −20 °C	R4 −20 °C	R1 −80 °C	R2 −80 °C	R3 −80 °C	R4 −80 °C
Results																										
		Albumin (g/dL) (3.5–5.0) *																								
**Mean concentration**		4.31	4.33	4.33	4.40	4.32	4.43	4.46	4.39	4.46	4.45	4.47	4.52	4.32	4.47	4.51	4.43	4.55	4.47	4.60	4.38	4.64	4.39	4.52	4.43	4.61
**Standard Deviation**		0.30	0.39	0.33	0.30	0.34	0.41	0.38	0.34	0.49	0.37	0.39	0.39	0.44	0.39	0.38	0.29	0.43	0.44	0.42	0.33	0.51	0.36	0.38	0.30	0.53
**Median**		4.28	4.31	4.25	4.35	4.28	4.38	4.36	4.31	4.32	4.41	4.37	4.39	4.27	4.40	4.41	4.38	4.39	4.39	4.47	4.34	4.46	4.30	4.42	4.39	4.43
**Range**		3.81–5.02	3.46–5.04	3.92–5.08	3.98–5.14	3.92–5.05	3.95–5.34	4.02–5.23	3.95–5.15	3.95–5.62	3.78–5.18	3.99–5.43	4.03–5.27	3.75–5.30	3.72–5.33	3.94–5.25	9.96–5.10	3.99–5.32	3.72–5.33	4.04–5.27	3.89–5.21	4.02–5.79	3.78–5.17	3.88–5.25	4.00–5.16	4.07–5.72
**Concentration percentage change (DB = 1.43%)**		0.00	0.46	0.52	2.13	0.17	2.64	3.38	1.94	3.37	3.09	3.56	4.81	0.26	3.71	4.70	2.72	5.45	3.76	6.78	1.59	7.52	1.72	4.82	2.67	6.91
***p* value**			0.666	0.627	**<0.05**	0.793	0.522	**<0.05**	**0.016**	0.350	**0.003**	0.057	**0.000**	0.794	**0.001**	**0.000**	**0.000**	**0.000**	**0.004**	**0.000**	**0.014**	**0.000**	0.079	**0.007**	**0.00**	**0.00**
		**Total Protein (g/dL)** (6.0–8.0) *																								
**Mean concentration**		6.79	6.78	6.89	6.87	6.72	6.85	6.82	6.87	6.86	7.15	7.14	7.23	6.73	6.93	7.09	7.09	7.04	6.99	7.04	7.05	7.22	6.84	7.01	7.17	7.08
**Standard Deviation**		0.41	0.36	0.40	0.37	0.49	0.43	0.53	0.36	0.38	0.44	0.41	0.42	0.36	0.43	0.38	0.46	0.45	0.45	0.44	0.49	0.44	0.38	0.45	0.51	0.42
**Median**		6.69	6.75	6.84	6.84	6.67	6.73	6.73	6.86	6.82	7.07	7.09	7.17	6.72	6.82	7.07	7.03	7.12	6.83	6.87	6.93	7.19	6.74	6.94	7.12	7.10
**Range**		6.11–7.83	6.24–7.73	6.21–7.82	6.23–7.86	5.89–7.99	6.17–7.85	6.14–8.14	6.18–7.82	6.34–7.81	6.38–8.25	6.56–8.22	6.63–8.25	5.96–7.50	6.36–7.91	6.49–8.15	6.43–8.31	6.39–8.06	6.00–8.15	6.52–8.25	6.44–8.33	6.40–8.39	6.32–7.95	6.43–8.30	6.44–8.54	6.41–8.21
**Concentration percentage change (DB = 1.36%)**		0.0	−0.18	1.37	1.20	−1.02	0.81	0.39	1.23	1.09	5.32	5.11	6.44	−0.83	2.04	4.38	4.41	3.68	2.99	3.68	3.85	6.40	0.73	3.29	5.59	4.29
***p* value**			0.808	0.067	**0.04**	0.091	0.186	0.852	0.073	0.575	**0.000**	**0.004**	**0.000**	0.184	0.056	**0.000**	**0.000**	**0.002**	**0.003**	**0.000**	**0.000**	**0.000**	**0.000**	**0.003**	**0.000**	**0.000**
		**Bilirubin (mg/dL)** (<1.3) *																								
**Mean concentration**		0.57	0.54	0.43	0.20	0.57	0.57	0.57	0.57	0.62	0.54	0.67	0.56	0.55	0.55	0.52	0.62	0.56	0.55	0.45	0.57	0.44	0.53	0.38	0.42	0.30
**Standard Deviation**		0.17	0.16	0.12	0.06	0.17	0.17	0.17	0.16	0.16	0.18	0.19	0.18	0.16	0.16	0.17	0.18	0.18	0.16	0.16	0.16	0.17	0.15	0.13	0.12	0.10
**Median**		0.58	0.54	0.41	0.20	0.57	0.57	0.56	0.57	0.61	0.54	0.68	0.57	0.55	0.57	0.52	0.61	0.58	0.54	0.44	0.54	0.42	0.53	0.37	0.40	0.27
**Range**		0.26–0.88	0.24–0.82	0.22–0.64	0.12–0.31	0.26–0.86	0.24–0.87	0.25–0.86	0.28–0.87	0.29–0.92	0.21–0.87	0.32–1.03	0.23–0.89	0.23–0.83	0.23–0.85	0.18–0.84	0.27–0.95	0.21–0.88	0.27–0.83	0.180–0.78	0.29–0.90	0.20–0.73	0.26–0.80	0.14–0.63	0.23–0.65	0.13–0.47
**Concentration percentage change (DB = 8.95%)**		00.0	−6.37	−25.87	−64.35	−0.61	−1.04	−1.63	−0.17	7.59	−5.88	17.49	−1.84	−4.25	−4.42	−8.79	8.72	−1.75	−3.56	−20.98	−0.96	−23.27	−6.93	−32.95	−26.16	−47.04
***p* value**			**0.000**	**0.000**	**0.000**	0.907	0.305	0.783	0.761	0.376	**0.000**	**0.022**	**0.018**	**0.000**	**0.001**	**0.000**	0.061	0.229	0.096	**0.000**	0.482	**0.003**	**0.000**	**0.000**	**0.000**	**0.000**
		Uric acid (mg/dL) (women < 5.7; men < 7.0) *																								
**Mean concentration**		4.72	4.76	4.85	4.87	4.74	4.79	4.74	4.61	4.75	4.88	4.87	4.88	4.72	4.74	4.91	4.82	4.85	4.85	4.92	4.87	4.94	4.81	4.85	4.83	4.85
**Standard deviation**		0.77	0.78	0.81	0.79	0.80	0.78	0.80	0.79	0.80	0.82	0.80	0.81	0.77	0.80	0.81	0.83	0.80	0.84	0.79	0.81	0.81	0.80	0.83	0.80	0.82
**Median**		4.89	4.83	4.96	4.99	4.89	4.96	4.86	4.57	4.95	5.08	4.99	5.03	4.90	4.81	5.07	4.97	4.97	4.99	5.03	4.99	5.12	4.95	5.08	5.02	5.01
**Range**		2.74–6.03	2.79–6.00	2.81–6.11	2.83–6.14	2.70–6.01	2.74–6.05	2.66–6.15	2.63–5.85	2.71–6.02	2.80–6.12	2.92–6.09	2.83–6.09	2.73–5.91	2.72–5.95	2.83–6.21	2.77–6.15	2.77–6.14	2.69–6.15	2.80–6.12	2.80–6.16	2.97–6.26	2.79–6.07	2.78–6.12	2.75–6.15	2.74–6.14
**Concentration percentage change (DB = 4.87%)**		0.00	0.78	2.56	3.13	0.26	1.35	0.35	−2.52	0.71	3.34	3.03	3.18	−0.07	0.23	4.02	2.10	2.69	2.61	4.13	2.99	4.66	1.76	2.79	2.69	2.80
***p* value**			0.297	0.311	**0.000**	0.750	0.077	0.815	**0.008**	0.777	**0.000**	0.116	**0.001**	0.921	0.848	**0.000**	0.451	**0.000**	0.364	**0.000**	**0.000**	0.383	**0.009**	**0.009**	**0.003**	**0.007**
		**FRAP (mmol/L)**																								
**Mean concentration**		1.14	1.11	1.07	1.03	1.16	1.17	1.12	1.19	1.11	1.19	1.48	1.05	1.22	1.20	1.35	1.04	1.25	1.07	1.29	1.44	1.00	1.19	1.27	1.11	1.19
**Standard deviation**		0.12	0.11	0.13	0.10	0.19	0.10	0.11	0.11	0.12	0.12	0.08	0.10	0.12	0.10	0.11	0.09	0.11	0.11	0.15	0.09	0.11	0.11	0.12	0.11	0.11
**Median**		1.16	1.13	1.08	1.04	1.18	1.20	1.13	1.22	1.14	1.23	1.49	1.06	1.25	1.21	1.36	1.04	1.25	1.08	1.32	1.44	1.02	1.22	1.30	1.11	1.18
**Range**		0.85–1.44	0.81–1.26	0.80–1.34	0.76–1.18	0.61–1.38	0.92–1.30	0.83–1.28	0.94–1.36	0.75–1.27	0.88–1.39	1.31–1.62	0.78–1.22	0.96–1.41	0.97–1.37	1.12–1.54	0.87–1.19	0.98–1.42	0.80–1.27	0.981–1.61	1.22–1.61	0.74–1.17	0.92–1.35	1.03–1.50	0.85–1.27	0.96–1.37
**Concentration percentage change (DB = 10%)**		0.00	−2.65	−6.50	−10.16	1.54	2.16	−2.45	4.71	−2.71	4.14	29.18	−7.89	6.57	4.94	18.27	−8.77	9.44	−6.21	12.60	26.12	−12.11	4.68	11.40	−2.91	4.64
***p* value**			**0.031**	**0.000**	**0.000**	**0.047**	**0.030**	**0.004**	**0.002**	**0.011**	**0.004**	**0.000**	**0.000**	**0.000**	**0.000**	**0.000**	**0.000**	**0.000**	**0.000**	**0.000**	**0.000**	**0.000**	**0.000**	**0.000**	**0.031**	**0.000**
		**AOPP (µmol/L)**																								
**Mean concentration**		58.80	59.14	61.10	48.86	58.25	43.90	55.22	59.09	57.80	65.45	68.43	59.18	48.53	62.45	66.42	62.78	56.16	50.52	100. 32	102. 32	108. 79	62.93	79.58	73.51	81.27
**Standard deviation**		11.48	10.74	11.15	11.29	11.50	14.46	13.74	17.21	16.29	16.20	16.11	16.33	13.78	14.95	16.63	18.74	26.61	17.21	31.03	38.16	34.45	13.88	24.42	26.61	33.69
**Median**		56.21	58.92	58.39	46.99	56.44	41.77	52.06	55.52	54.91	62.57	63.51	54.19	45.57	59.79	60.73	58.88	63.49	55.52	90.89	91.67	100.61	59.89	73.13	63.49	73.44
**Range**		47.23–101.24	44.75–93.94	49.43–100. 96	33.30–86.88	44.15–99.50	23.97–85.89	44.75–107.87	44.69–121.99	40.46–116.52	51.05–125.31	53.51–127.84	43.08–119.64	40.46–116.52	48.48–119.50	51.08–126.71	44.46–130.44	37.71–121.92	32.86–114.72	68.36–161.45	62.70–210.51	64.40–191.95	48.94–109.97	52.83–149.05	42.60–137.50	46.25–173.91
**Concentration percentage change (DB = 10%)**		0.00	0.57	3.90	−16.91	−0.94	−25.34	−6.09	0.50	−1.69	11.32	16.38	0.65	−17.46	6.22	12.97	6.78	−4.49	−14.07	70.61	74.02	85.03	7.00	35.34	25.02	38.21
***p* value**			0.765	**0.012**	**0.000**	0.550	**0.000**	**0.002**	0.910	0.525	**0.000**	**0.000**	0.736	**0.000**	**0.017**	**0.000**	0.079	0.178	**0.011**	**0.000**	**0.000**	**0.000**	**0.009**	**0.000**	**0.004**	**0.001**

DB—desirable bias [19]. values above DB are shown in gray cells; T_0_–baseline; RT 24–120 h—values of analyzed parameters after storage of serum samples for 24–120 h at room temperature; FT 24–120 h—values of analyzed parameters after storage of serum sample for 24–120 h at 4 °C; 1M–6M—values of analyzed parameters after storage of serum samples for 1–6 months at (−20 °C) and (−80 °C); R1–R4—values of analyzed parameters after cyclic defrosting of serum after storage at −20 °C and −80 °C; FRAP—ferric reducing ability of plasma; AOPP—advanced oxidation protein products; * reference range. Information about reference ranges for parameters studied were obtained from [29]. A two-tailed *p*-value of less than 0.05 was considered to be significant (marked in bold font).

## Supplementary Materials

Supplementary materials can be found at https://www.mdpi.com/2075-4418/10/1/51/s1.
