# Supplementary material for: The Influence of Serum Sample Storage Conditions on Selected Laboratory Parameters Related to Oxidative Stress: A Preliminary Study
Source: Diagnostics (Basel). 2020 Jan 19;10(1):51. doi: 10.3390/diagnostics10010051 (PMC7168228; doi:10.3390/diagnostics10010051)
Supplement: Supplementary file 1 [file diagnostics-10-00051-s001.pdf]

diagnostics

**Analysis of the dependence of variability of determined parameters on their initial concentration**

In purpose of assessing potential influence of initial concentration of tested in this study parameters coefficient of variation (CV) for every patient was calculated. First the standard deviation (SD) of results obtained for patient samples stored under specific condition (room temperature (3 results), refrigerator (3 results), −20 °C (6 results) −80 °C (6 results) and the same freezer temperature with cyclic thawing/freezing (4 results for −20 °C and 4 results for −80 °C) were calculated. Next, calculated SD was divided by concentration in T0 measured for this patient and multiplied by 100 in order to express the results as a percentage value.

The results were illustrated on the Figures 1–6 when on the x-axis are placed observed initial concentrations of analysed parameters (T0) (each specific concentration correspond to the subsequent analysed patient) and on the y-axis are placed values of CV observed for each patient sample stored in specific conditions pointed out on the chart legend.

On the figures 1 −6 the results of each analysed parameters were divided on three panels for increasing the readability of the results: panel "A" is dedicated for short term storage, panel "B" for samples stored frozen in two different temperatures and panel "C" for samples stored frozen in two different temperatures, but additionally thawing and freezing again from one to four times. In the next step the correlations of CV in function of initial parameters' concentrations were calculated. The Spearman correlation test was used because of non-Gaussian distribution of observed CV in all groups and the results are showed in Table 1.

For the short time of storage in room temperature for bilirubin and uric acid we observed the significant correlation, when the dispersion of observed results correlates with the increase in the initial concentration of bilirubin and uric acid, what means that for the samples with a higher concentration, a greater change in concentration can be expected. For refrigerator temperature such correction was observed only for AOPP.

When we analyzed long time period of storage, the correlation of CV and initial concentration was observed for the total protein and FRAP in −20 °C and for AOPP in −80 °C.

When the long-time storage in freezer was combined with thawing and freezing cycles it affected albumin concentration in both freezing temperatures, what was not observed for this parameter in simple storage in −20 °C and −80 °C. A similar effect was observed for bilirubin, when CV value did not correlate with initial concentration for storing in −80 °C, but thawing and freezing cycles in the same temperature points to the relation between CV and initial concentration. For AOPP concentration the cycling thawing and freezing deepened the correlation of CV and initial concentration observed for samples stored in −80 °C.

In our opinion the observed coefficient of correlation, even though significant, indicated weak or moderated correlation ("r" from 0.274 to 0.543). The high correlations were observed for bilirubin concentration after storage in room temperature, what is a well-known fact. For albumin, bilirubin and AOPP, extremely unfavourable condition of repeated thawing and freezing as well as long term storing in freezer before total protein and AOPP examination we also observed high correlation of CV with initial concentration.

Our main analysis presented in manuscript exclude long storage in freezing condition for protein related parameters, therefore the results discussed above were not included to the discussion section.

Our results in regards to AOPP stay in a visible contradiction to the literature data (Witko-Sarsat V, Friedlander M, Capeillère-Blandin C, Nquyen-Khoa T, Nquyen AT, Zingraff J et al. Advanced oxidation protein products as a novel marker of oxidative stress in uremia. Kidney Int. 1996; 49:1304–1313), but the authors examined smaller group of subjects to which were included various participants, who also differ in AOPP concentration (Witko-Sarsat documented that: "AOPP levels were significantly higher in plasma of HD patients (N10) than of controls: undialyzed patients with advanced renal failure ($n$ = 10;137.6 ± 11.1 vs. 29.4 ± 4.9μmol/litre"). Witko-Sarsat et al. doesn't shown the stability and storage condition of samples and criteria used by authors are not mentioned (they

concluded that samples can be stored in −70 °C during 6 months, but no other temperature of storage was investigated), thus we are not able to discuss about it in detail.

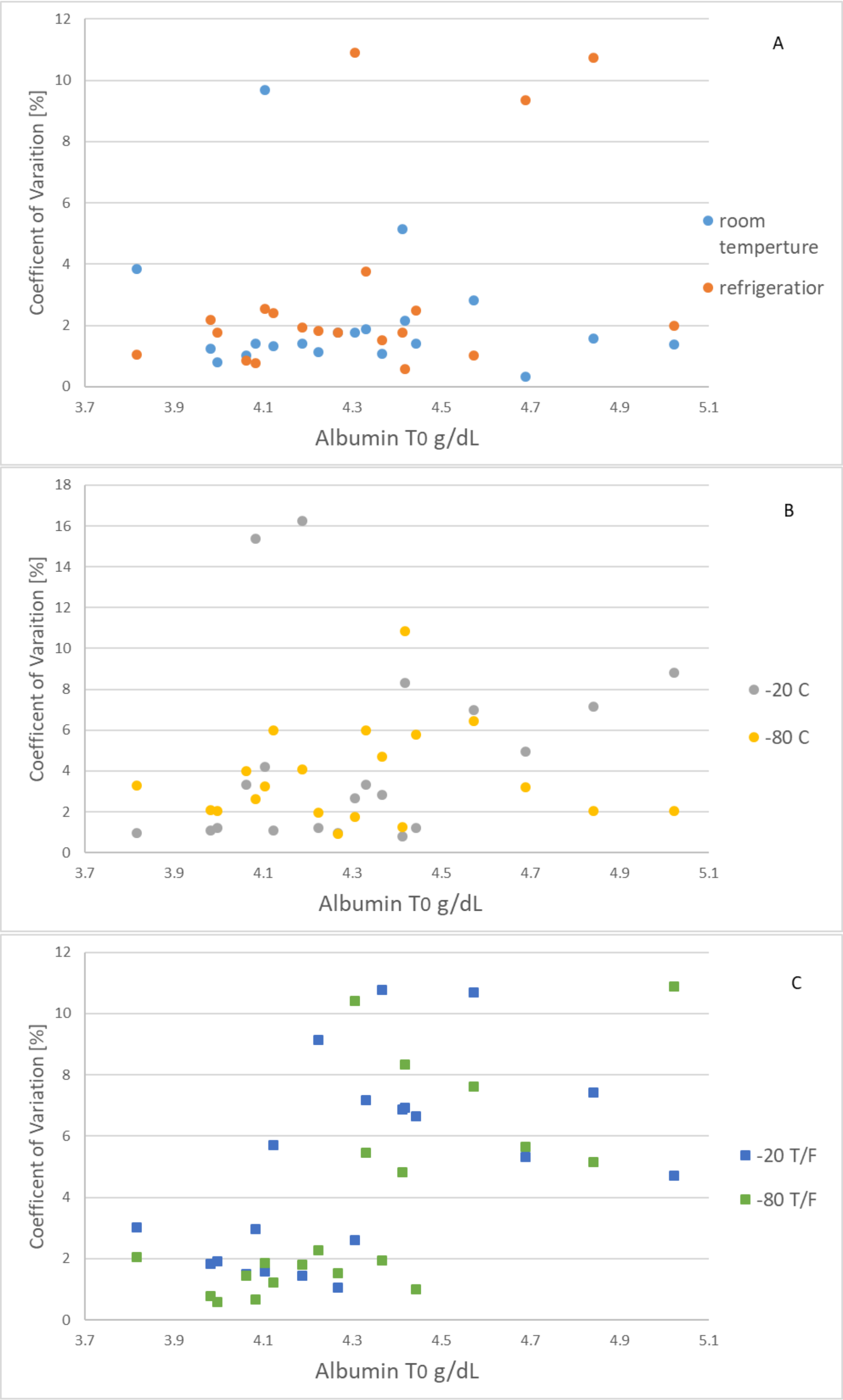


**Figure 1.** Plot of the coefficient of variation for determinations carried out in samples stored in RT and +4 °C (panel A), −20 °C and −80 °C (panel B) and thawing and freezing (T/F) cycles in −20 °C and −80 °C (panel C) depending on the initial concentration (T0) of albumin.

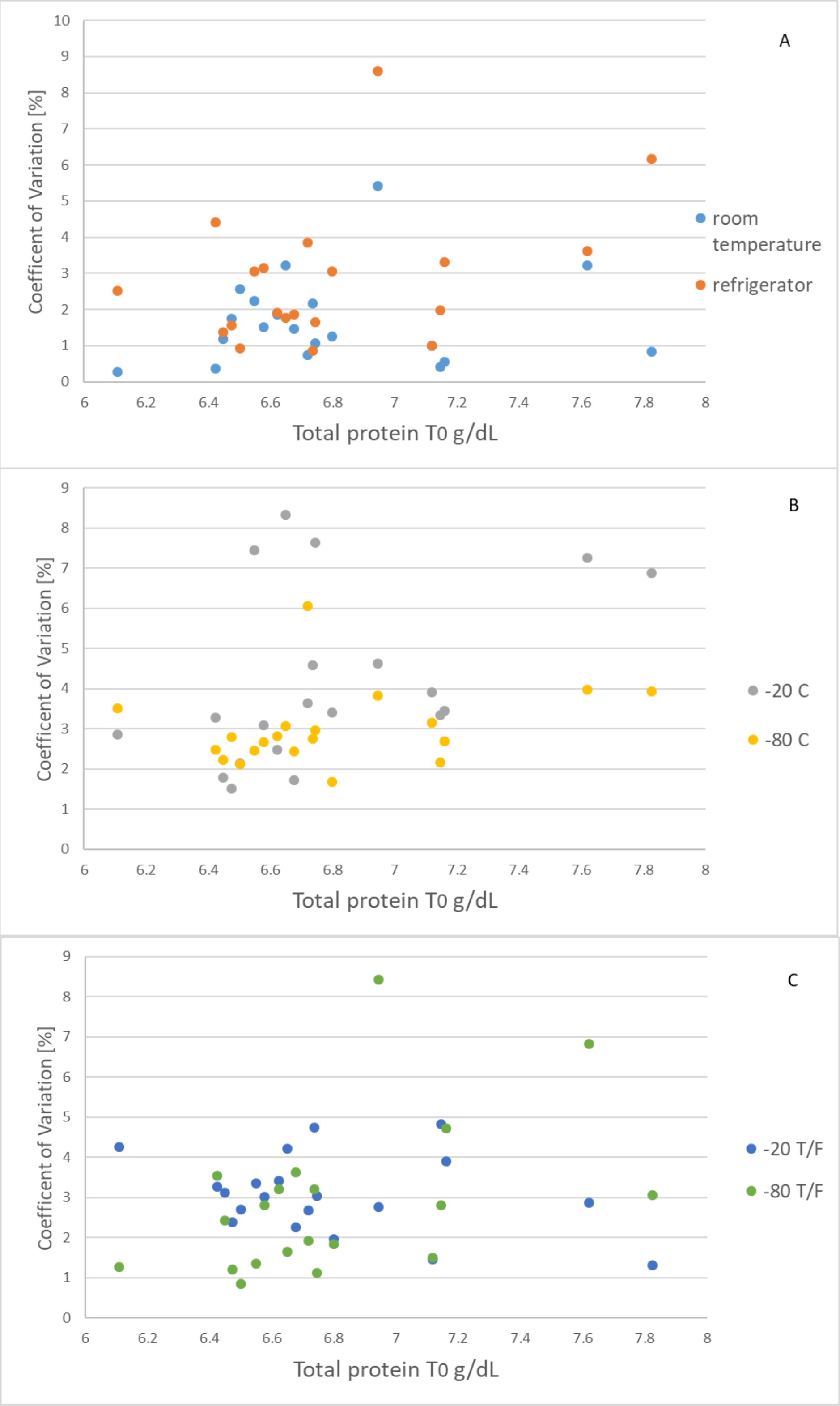


**Figure 2.** Plot of the coefficient of variation for determinations carried out in samples stored in RT and +4 °C (panel A), −20 °C and −80 °C (panel B) and thawing and freezing (T/F) cycles in −20 °C and −80 °C (panel C) depending on the initial concentration (T0) of total protein.

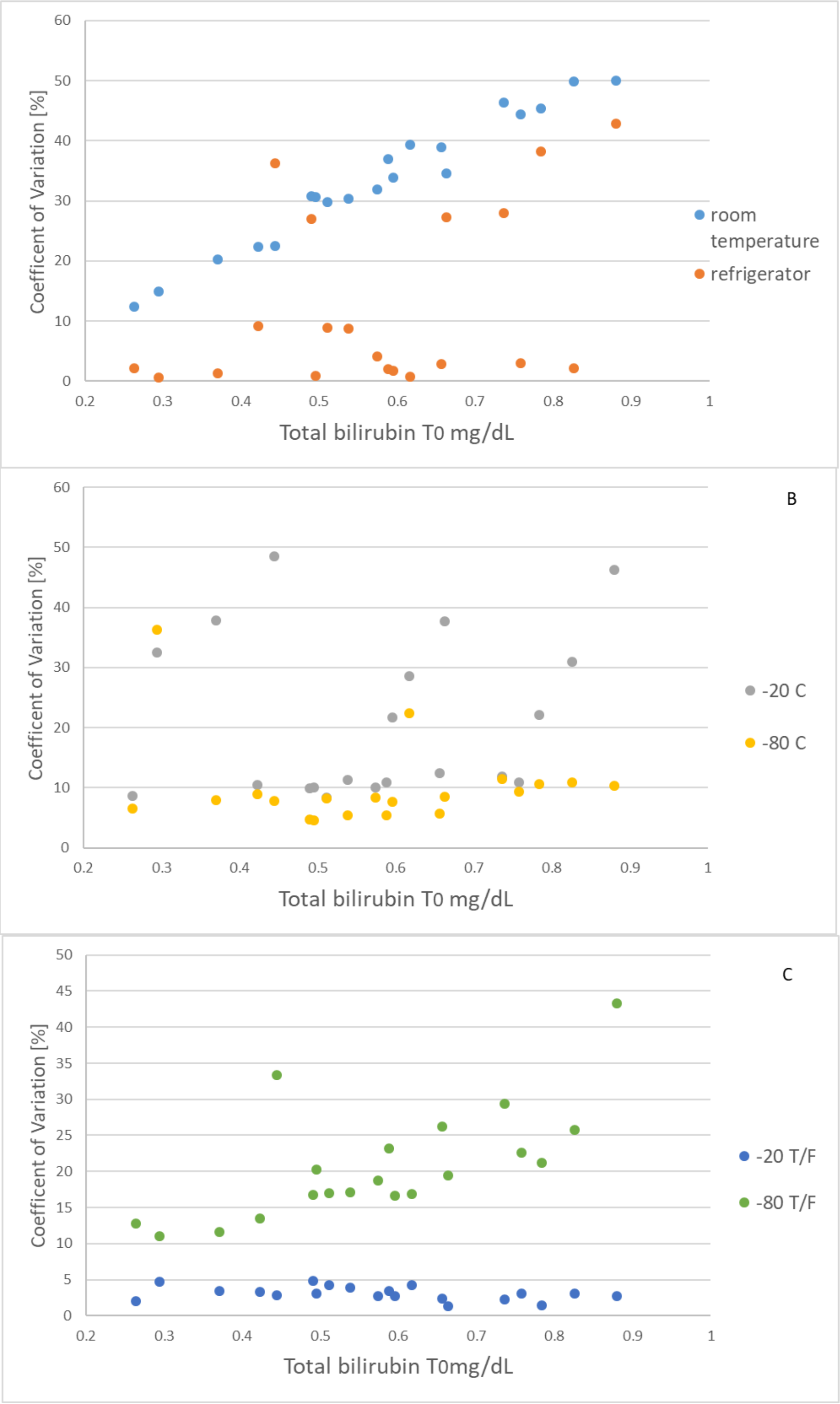


**Figure 3.** Plot of the coefficient of variation for determinations carried out in samples stored in RT and +4 °C (panel A), −20 °C and −80 °C (panel B) and thawing and freezing (T/F) cycles in −20 °C and −80 °C (panel C) depending on the initial concentration (T0) of total bilirubin.

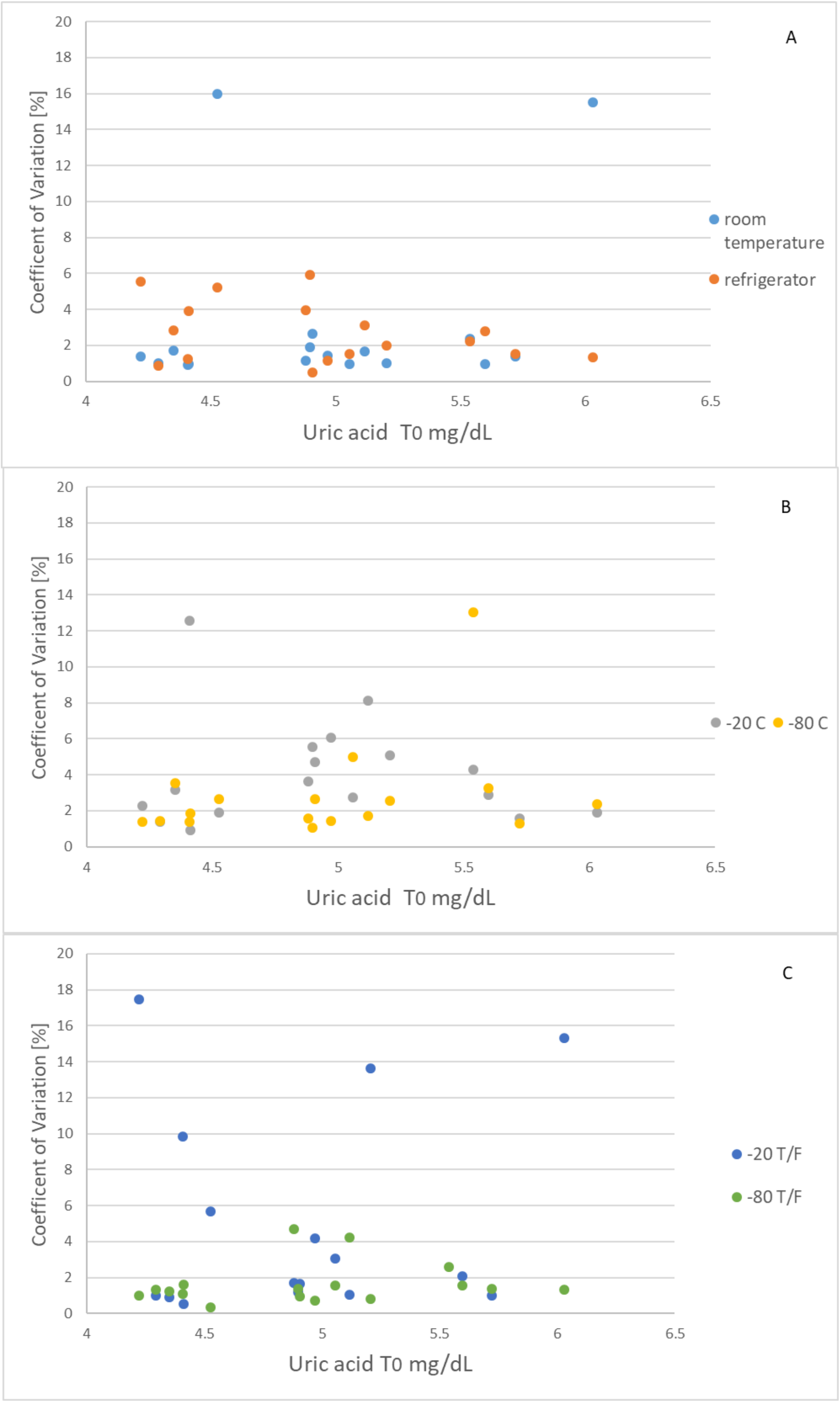


**Figure 4.** Plot of the coefficient of variation for determinations carried out in samples stored in RT and +4 °C (panel A), −20 °C and −80 °C (panel B) and thawing and freezing (T/F) cycles in −20 °C and −80 °C (panel C) depending on the initial concentration (T0) of uric acid.

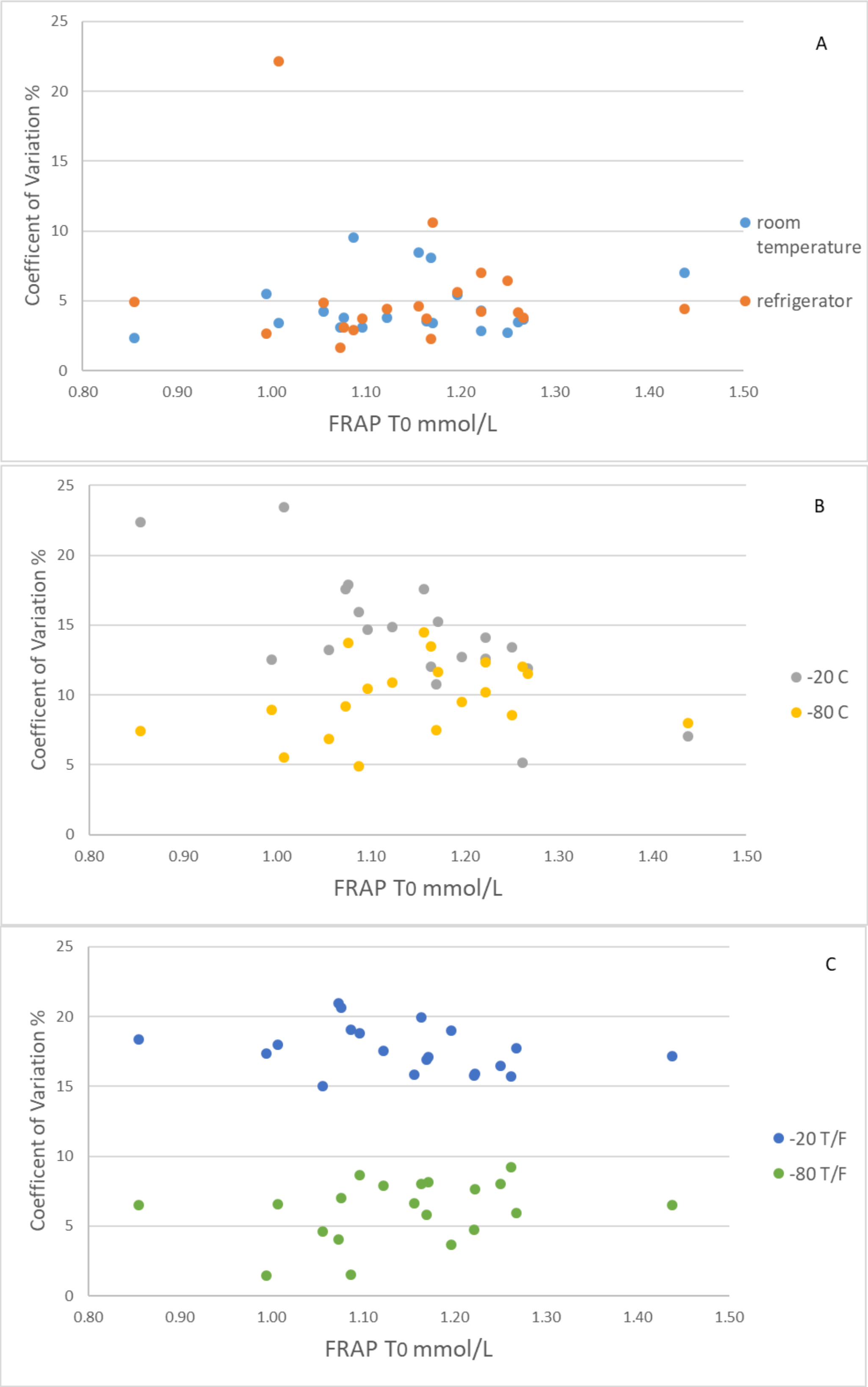


**Figure 5.** Plot of the coefficient of variation for determinations carried out in samples stored in RT and +4 °C (panel A), −20 °C and −80 °C (panel B) and thawing and freezing (T/F) cycles in −20 °C and −80 °C (panel C) depending on the initial concentration (T0) of FRAP.

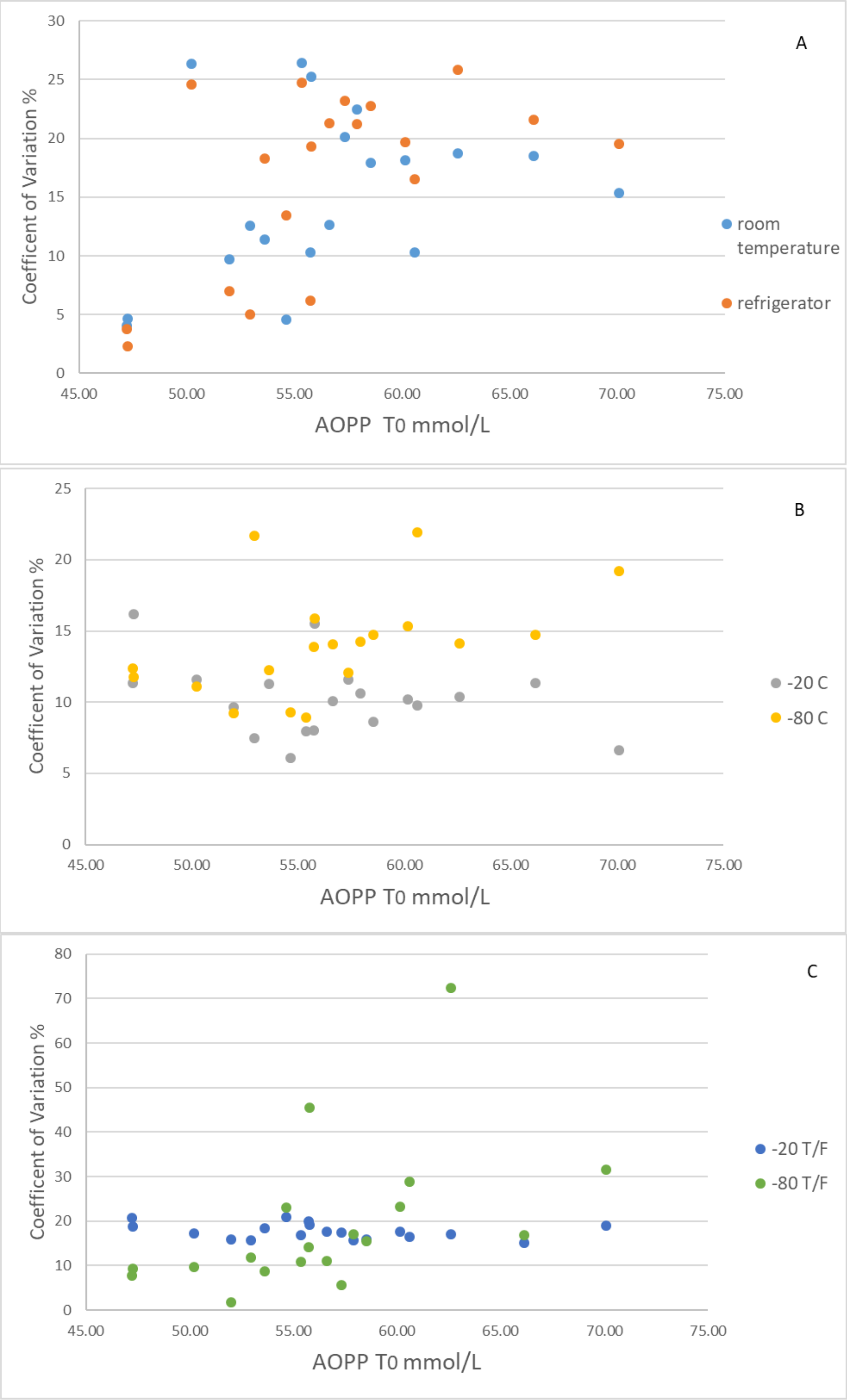


**Figure 6.** Plot of the coefficient of variation for determinations carried out in samples stored in RT and +4 °C (panel A), −20 °C and −80 °C (panel B) and thawing and freezing (T/F) cycles in −20 °C and −80 °C (panel C) depending on the initial concentration (T0) of AOPP.

**Table 1.** Correlation of coefficient of variation (CV) calculated for specific storage condition with initial concentration ($T_0$) of analyzed parameters.

| Parameter | Storage condition | Spearman correlation (r) | *p* |
|---|---|---|---|
| **Albumin** | RT | 0.107 | 0.654 |
| | +4 °C | 0.296 | 0.205 |
| | −20 °C | 0.371 | 0.107 |
| | −80 °C | 0.086 | 0.719 |
| | −20 °C T/F | 0.274 | 0.008 |
| | −80 °C T/F | 0.689 | <0.001 |
| **Total Protein** | RT | 0.019 | 0.934 |
| | +4 °C | 0.300 | 0.197 |
| | −20 °C | 0.543 | 0.013 |
| | −80 °C | 0.311 | 0.182 |
| | −20 °C T/F | −0.230 | 0.329 |
| | −80 °C T/F | 0.439 | 0.053 |
| **Bilirubin** | RT | 0.968 | <0.000 |
| | +4 °C | 0.347 | 0.133 |
| | −20 °C | 0.299 | 0.200 |
| | −80 °C | 0.388 | 0.091 |
| | −20 °C T/F | 0.229 | 0.333 |
| | −80 °C T/F | 0.699 | <0.001 |
| **Uric Acid** | RT | 0.481 | 0.032 |
| | +4 °C | −0.003 | 0.990 |
| | −20 °C | −0.061 | 0.796 |
| | −80 °C | 0.272 | 0.246 |
| | −20 °C T/F | −0.105 | 0.659 |
| | −80 °C T/F | 0.350 | 0.130 |
| **FRAP** | RT | 0.062 | 0796 |
| | +4 °C | 0.183 | 0.439 |
| | −20 °C | −0.657 | 0.002 |
| | −80 °C | 0.353 | 0.126 |
| | −20 °C T/F | −0.383 | 0.095 |
| | −80 °C T/F | 0.316 | 0.175 |
| **AOPP** | RT | 0.371 | 0.117 |
| | +4 °C | 0.514 | 0.024 |
| | −20 °C | −0.203 | 0.403 |
| | −80 °C | 0.609 | 0.006 |
| | −20 °C T/F | 0.310 | 0.196 |
| | −80 °C T/F | 0.712 | <0.001 |

AOPP - Advanced Oxidation Protein Products; FRAP - Ferric Reducing Ability of Plasma; RT – room temperature; T/F – cycling of thawing and re-freezing of the same samples; T0 - initial concentrations measured in the day of blood sampling. Two-tailed *p*-value of less than 0.05 was considered to be significant (marked in red).

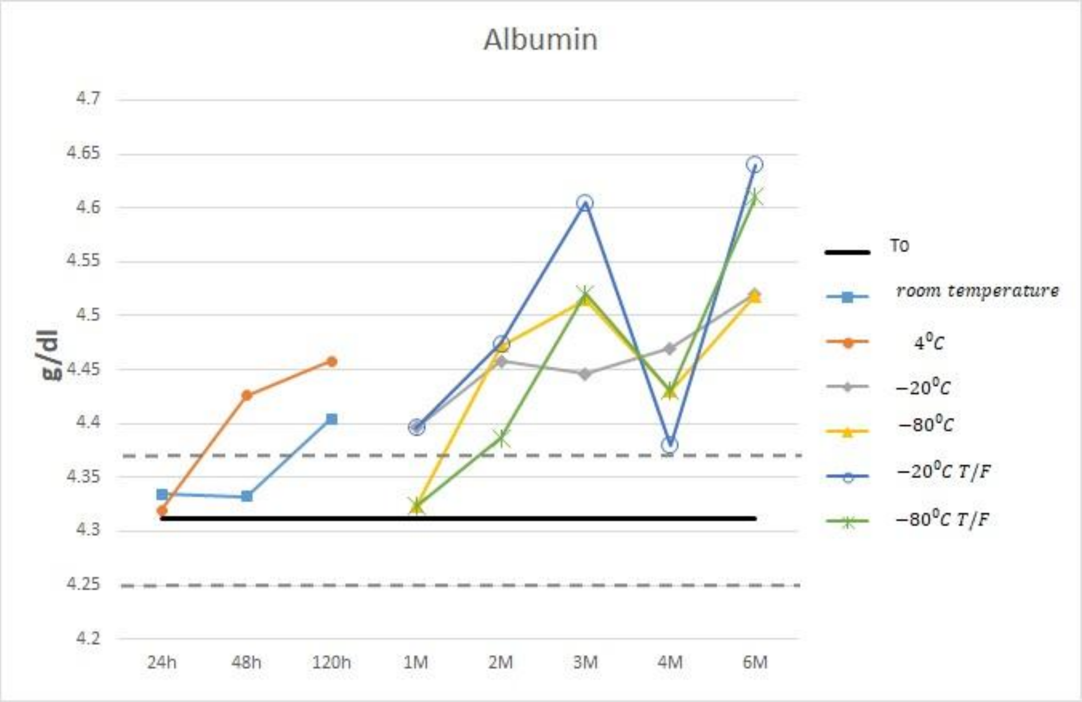


**Figure 7.** Changes in concentration of Albumin after storage of serum samples in dependency on time and temperature; T0 - initial concentration, T/F – cycling of thawing and re-freezing of the same samples; Desirable Bias is marked as dotted line.

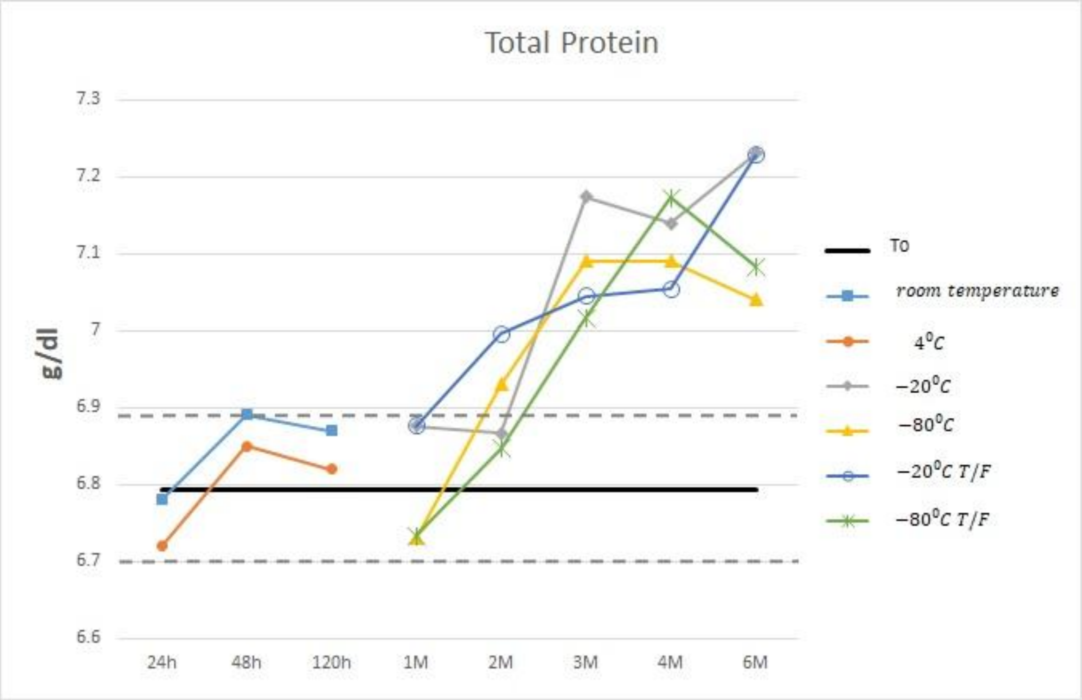


**Figure 8.** Changes in concentration of Total Protein after storage of serum samples in dependency on time and temperature; T0 - initial concentration, T/F – cycling of thawing and re-freezing of the same samples; Desirable Bias is marked as dotted line.

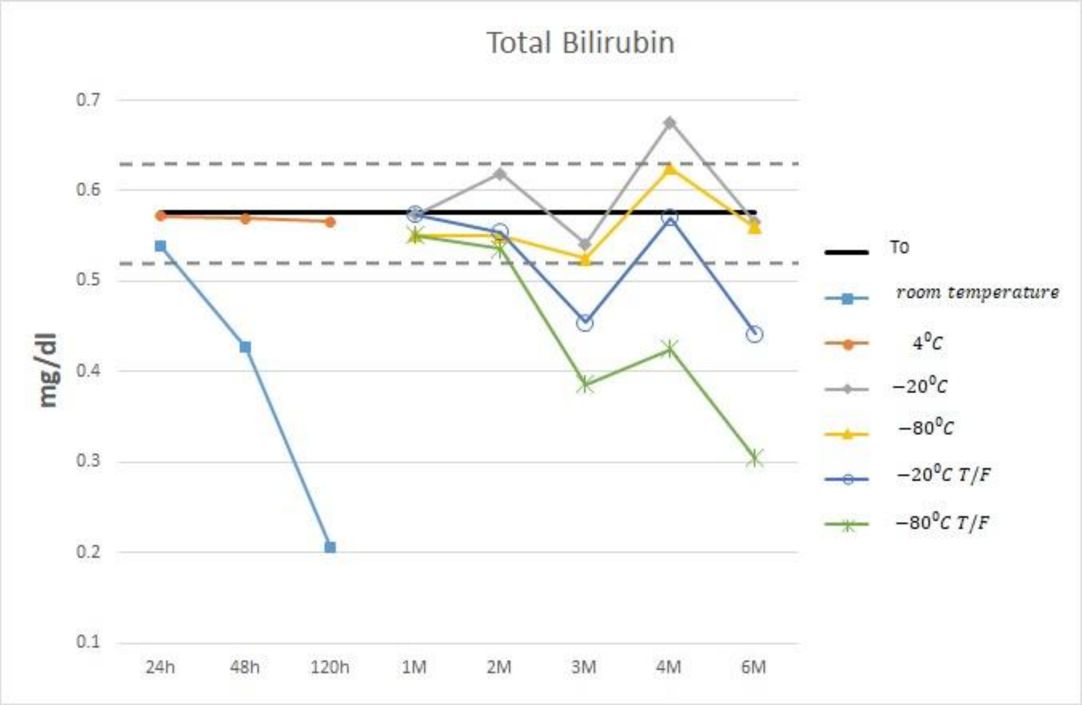


**Figure 9.** Changes in concentration of Total bilirubin after storage of serum samples in dependency on time and temperature; T0 - initial concentration, T/F – cycling of thawing and re-freezing of the same samples; Desirable Bias is marked as dotted line.

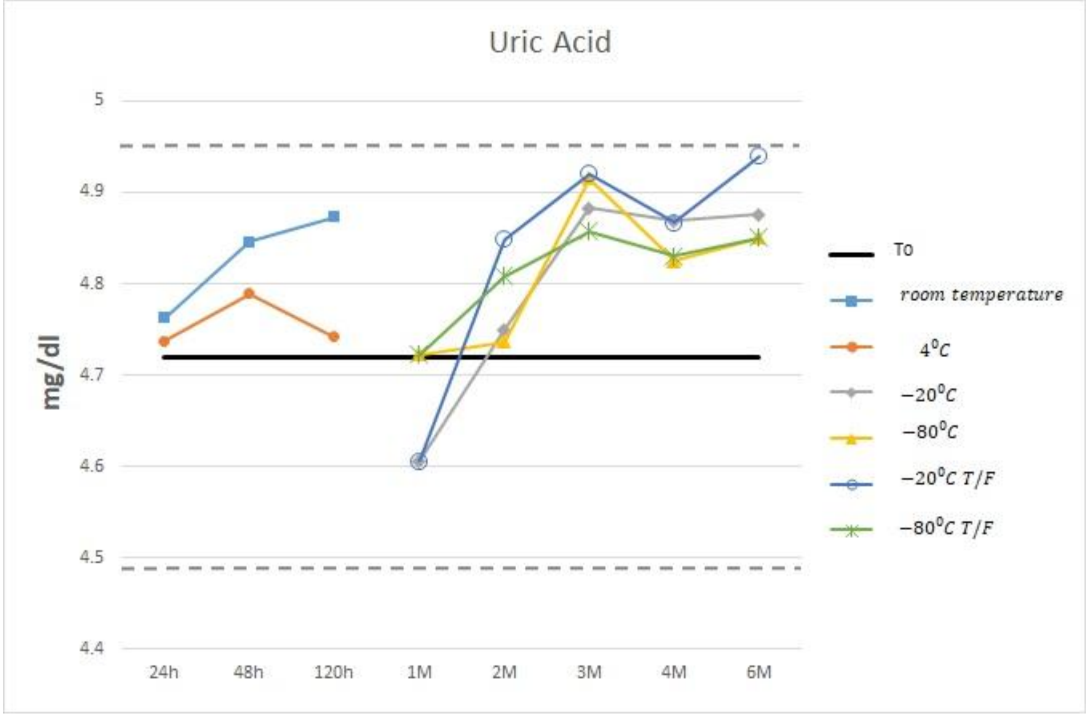


**Figure 10.** Changes in concentration of Uric acid after storage of serum samples in dependency on time and temperature; T0 - initial concentration, T/F – cycling of thawing and re-freezing of the same samples; Desirable Bias is marked as dotted line.

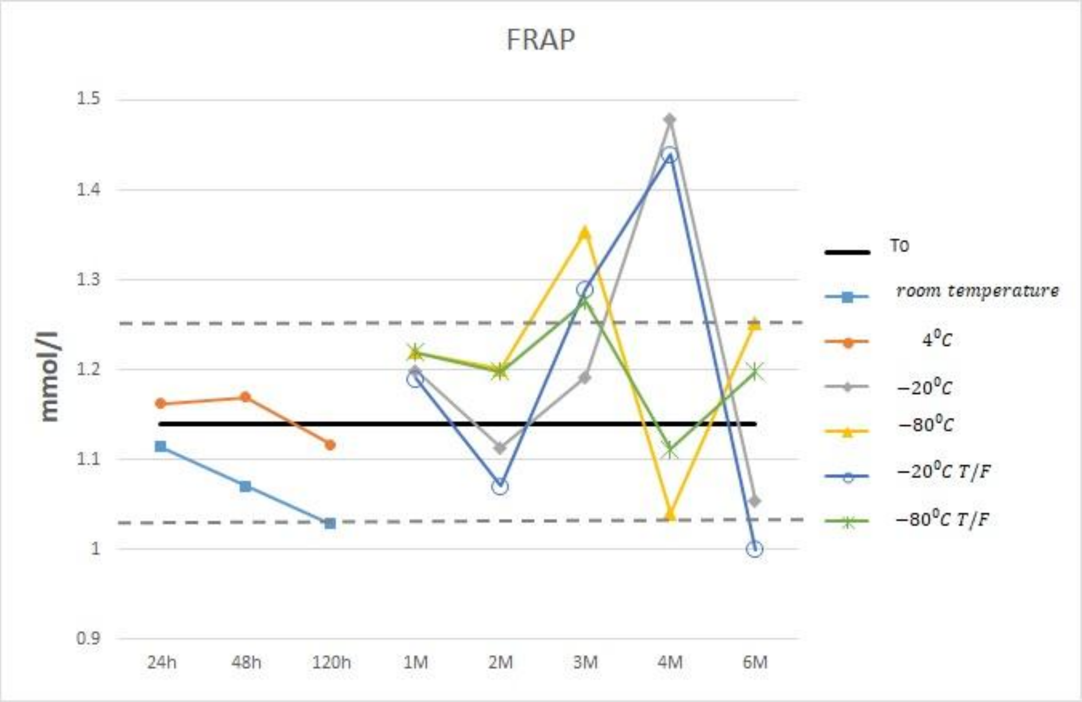


**Figure 11.** Changes in concentration of FRAP after storage of serum samples in dependency on time and temperature; T0 - initial concentration, T/F – cycling of thawing and re-freezing of the same samples; Desirable Bias is marked as dotted line.

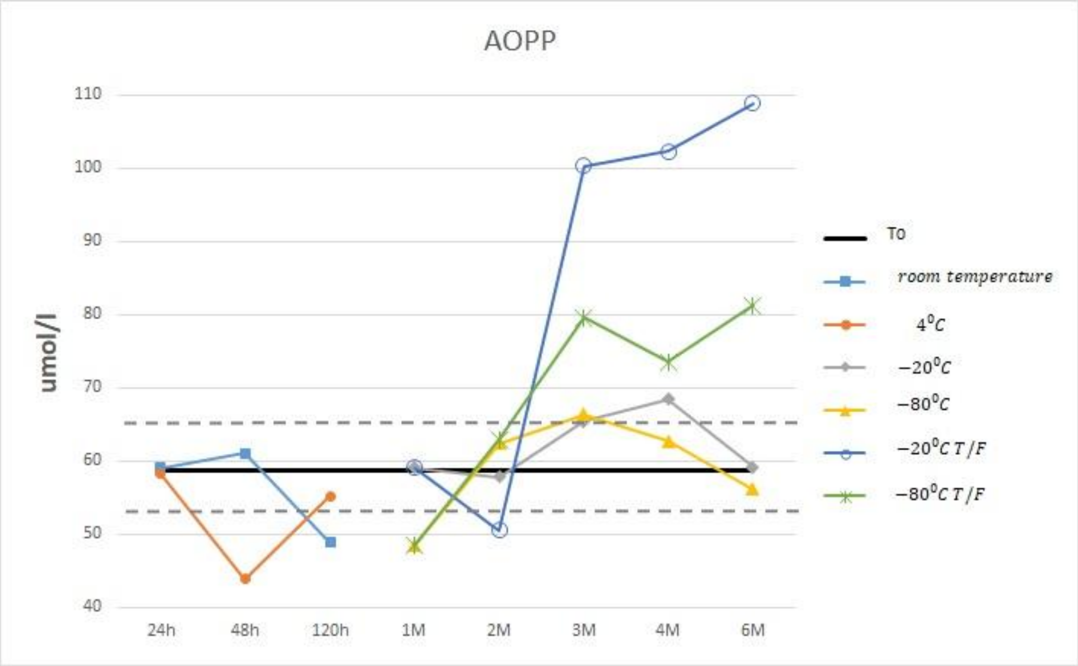


**Figure 12.** Changes in concentration of AOPP after storage of serum samples in dependency on time and temperature; T0 - initial concentration, T/F – cycling of thawing and re-freezing of the same samples; Desirable Bias is marked as dotted line.

© 2020 by the authors. Licensee MDPI, Basel, Switzerland. This article is an open access article distributed under the terms and conditions of the Creative Commons Attribution (CC BY) license (http://creativecommons.org/licenses/by/4.0/).
